# Bilateral Pheochromocytoma in a Child Revealing Von Hippel-Lindau Disease

**DOI:** 10.7759/cureus.84781

**Published:** 2025-05-25

**Authors:** Nadia Echcharii, Nabila Chekhlabi, Amal Rami, Amal Miqdadi, Nezha Dini

**Affiliations:** 1 Pediatrics, Cheikh Khalifa International University Hospital, Mohammed VI University of Health Sciences, Casablanca, MAR; 2 Radiology, Cheikh Khalifa International University Hospital, Mohammed VI University of Health Sciences, Casablanca, MAR; 3 Nuclear Medicine, Cheikh Khalifa International University Hospital, Mohammed VI University of Health Sciences, Casablanca, MAR

**Keywords:** bilateral adrenal tumor, genetic testing, pediatric hypertension, pheochromocytoma, von hippel-lindau disease

## Abstract

We report the case of a 14-year-old boy admitted with malignant hypertension, headaches, vomiting, and generalized seizures. Clinical, biochemical, and imaging evaluations revealed bilateral adrenal masses with elevated plasma metanephrines, consistent with bilateral pheochromocytoma. After preoperative preparation with an alpha-blocker, the patient underwent right adrenalectomy, and histopathological analysis confirmed a low-aggressive pheochromocytoma. Postoperatively, his blood pressure normalized, catecholamine levels returned to normal, and cardiac function improved. Genetic testing identified a pathogenic variant in the von Hippel-Lindau (VHL) gene, confirming the diagnosis of VHL disease. A 14-month follow-up showed clinical stability, no recurrence, and no additional tumors during systematic screening. This case highlights the importance of early diagnosis and genetic screening in pediatric pheochromocytoma, especially in bilateral cases, to guide treatment and long-term surveillance.

## Introduction

Pheochromocytoma is a rare neuroendocrine tumor that originates from chromaffin cells of the adrenal medulla. In the pediatric population, pheochromocytomas are extremely rare, with an estimated incidence of 0.2-0.5 cases per million children per year. Although typically benign, it causes excessive catecholamine secretion, leading to severe and potentially life-threatening hypertension [1]. Diagnosis relies on biochemical assays, including plasma and urinary metanephrines, and imaging techniques such as CT scans, MRI, and functional imaging (scintigraphy or PET scan). Bilateral pheochromocytomas account for less than 10% of cases and often have a genetic origin, notably associated with syndromes such as von Hippel-Lindau (VHL) disease [1]. Bilateral pheochromocytomas are even less common and are usually a clinical clue pointing toward an underlying genetic predisposition. Since approximately 30% of pheochromocytomas are hereditary, genetic screening is crucial in patient management and long-term follow-up [2]. We present the case of a 14-year-old adolescent diagnosed with bilateral adrenal pheochromocytoma, ultimately leading to the identification of VHL disease. This report highlights the diagnostic approach, therapeutic strategies, and the importance of genetic screening in pediatric pheochromocytomas.

## Case presentation

We present the case of a 14-year-old boy admitted for malignant hypertension, initially manifesting in November 2023 with headaches, vomiting, and generalized tonic-clonic seizures lasting several minutes, interpreted as status epilepticus. Despite initial treatment, hypertension persisted, leading to his referral to our center for specialized care. The patient had no history of chronic illnesses, with normal psychomotor development and appropriate growth parameters. His family history revealed a paternal aunt with breast cancer and a grandfather with colon cancer. No consanguinity was reported, and his siblings were in good health.

Upon admission, the patient weighed 32 kg (-1 SD) and measured 145 cm (+0.5 SD), with severely elevated blood pressure (215/138 mmHg) and tachycardia (125 bpm). His physical examination showed stable respiratory status, no signs of heart failure, and a soft abdomen without palpable masses. No café-au-lait spots were observed. Initial investigations revealed a normal urinary profile, without proteinuria or hematuria. A fundoscopic examination revealed bilateral stellate macular edema with crowned nodules and exudates. Although an EEG could have been considered in the setting of seizures, it was not performed due to the urgency of blood pressure control and the endocrine workup prompted by the clinical picture. A chest X-ray was unremarkable, but echocardiography revealed left ventricular hypertrophy with a borderline ejection fraction of 58%. Given the neurological presentation and the risk of central nervous system tumors in VHL disease, a brain MRI was performed, which showed no abnormalities.

**Figure 1:**
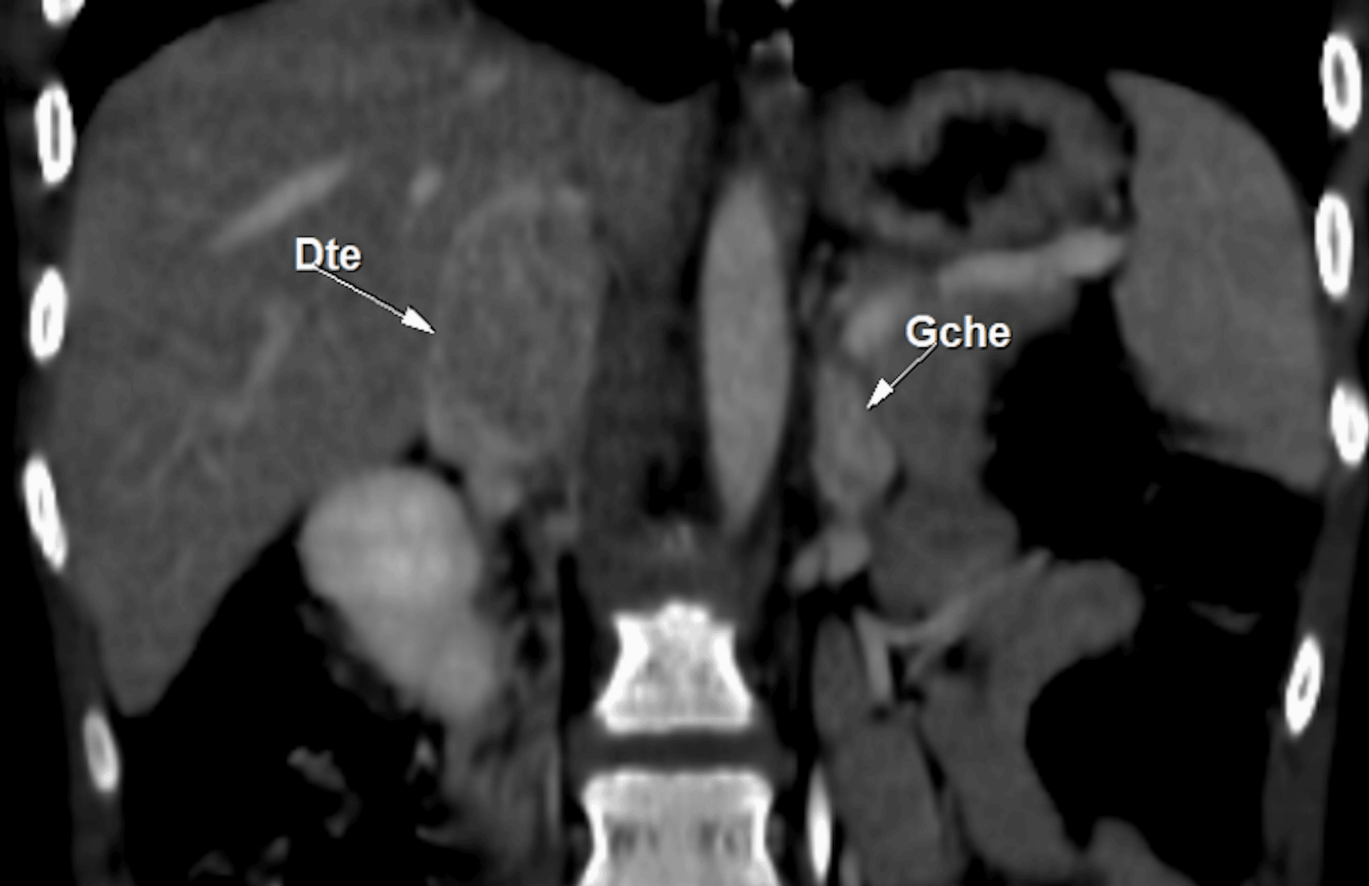
Coronal CT scan showing bilateral adrenal masses right: 3.5 × 2.5 cm; left: 13 × 12 mm CT: computed tomography

**Figure 2:**
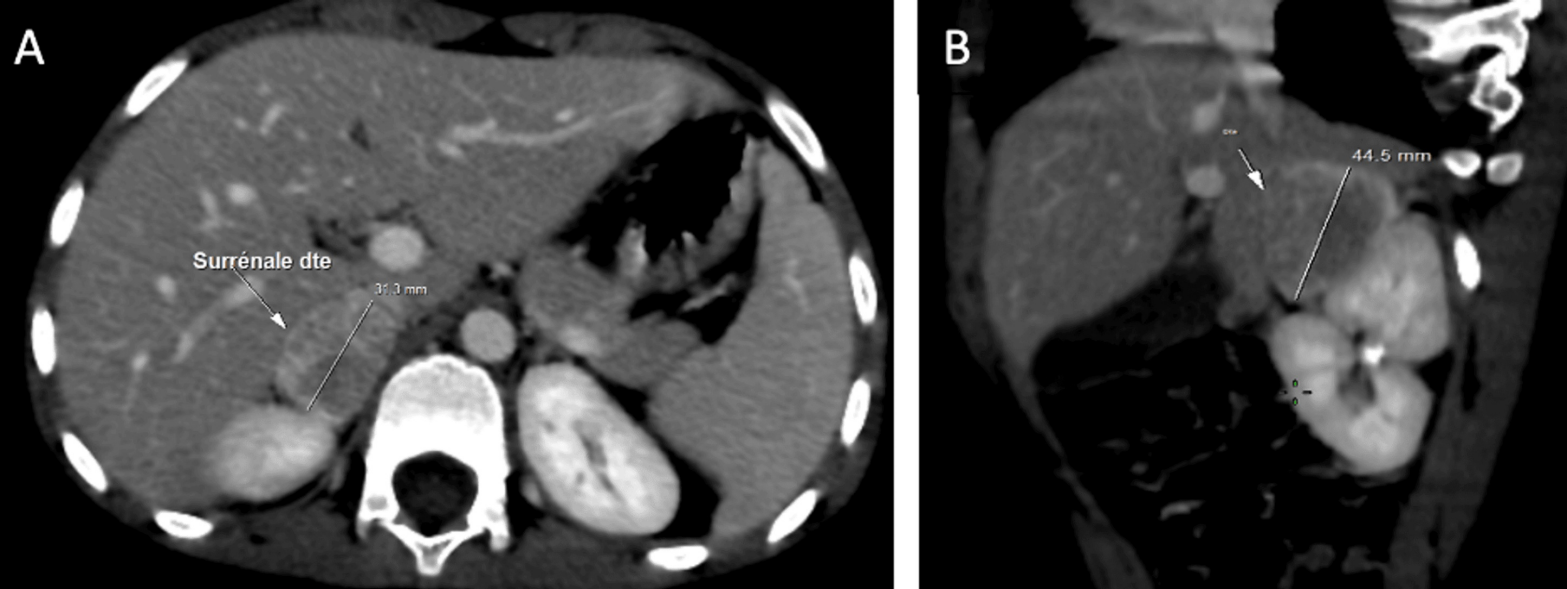
Axial (A) and parasagittal (B) views of the right adrenal gland (A) Axial view showing a right adrenal mass measuring 31.3 mm. (B) Parasagittal view showing the same hypervascularized mass measuring 44.5 mm.

**Figure 3:**
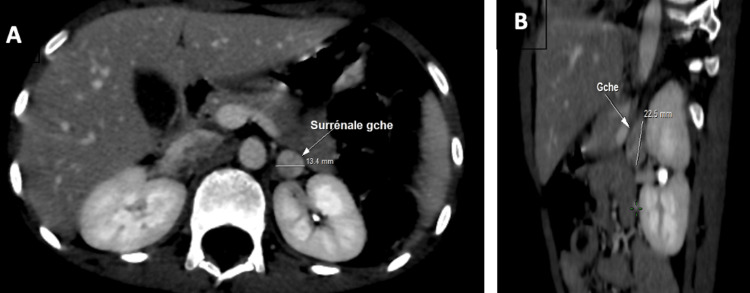
Axial (A) and coronal (B) views of the left adrenal gland (A) Axial view showing a left adrenal mass measuring 13.4 mm. (B) Coronal view showing the same hypervascularized mass, measuring 22.5 mm.

Abdominal CT imaging identified bilateral adrenal masses measuring 3.5 × 2.5 cm on the right and 13 × 12 mm on the left. These masses were hypervascularized and highly suggestive of bilateral pheochromocytoma (Figures 1-3).

**Figure 4:**
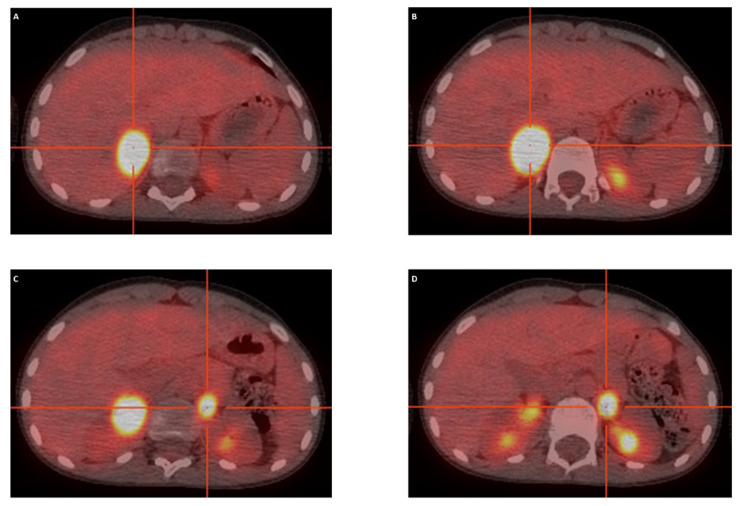
Axial PET/CT images showing bilateral adrenal hypermetabolism (A–D) PET/CT slices confirming intense bilateral adrenal FDG uptake without evidence of distant metastasis. PET/CT: positron emission tomography/computed tomography, FDG: fluorodeoxyglucose

PET imaging confirmed bilateral adrenal hypermetabolism with no evidence of metastasis (Figure 4).

Laboratory findings showed normal renal and adrenal function but markedly elevated plasma-free metanephrines (normetanephrine: 478 ng/L, metanephrine: 288 ng/L).

The patient was initially managed with continuous infusion of the calcium channel blocker nicardipine for blood pressure stabilization. To preserve adrenal function and avoid lifelong steroid dependence, a right unilateral adrenalectomy was performed following one week of preparation with the selective alpha-blocker doxazosin (4 mg/day). Histopathological analysis confirmed pheochromocytoma with a PASS score of 1, indicating low aggressiveness and no extra-adrenal extension.

The postoperative course in the intensive care unit was favorable, with rapid stabilization of vital parameters and normalization of blood pressure without needing antihypertensive medication. A 24-hour ambulatory blood pressure monitoring on the 10th postoperative day confirmed stable normotension. Serial echocardiograms demonstrated progressive regression of left ventricular hypertrophy and improved ejection fraction. Plasma and urinary catecholamine levels returned to normal: plasma-free normetanephrine: 202 pg/mL; urinary metanephrine: 0.04 mg/day; urinary normetanephrine: 0.32 mg/day (Table 1).

**Table 1:** Evolution of clinical and biological parameters before and after surgery Evolution of clinical and biochemical parameters before and after surgery. Measurements were taken at diagnosis (day 0) and postoperative day 10. "Not measured" indicates data not collected at the respective time point. Normal reference ranges are provided for pediatric patients.

Parameter	Day 0 (preoperative)	Day 10 (postoperative)	Normal range
Blood pressure (mmHg)	215/138	100/70	<110/75
Heart rate (bpm)	125	90	70-100
Plasma metanephrine (pg/mL)	478	202	<90
Plasma normetanephrine (pg/mL)	288	202	<180
Urinary metanephrine (mg/24 h)	Not measured	0.04	<0.1
Urinary normetanephrine (mg/24 h)	Not measured	0.32	<0.3

Genetic testing was conducted due to the bilateral nature of the pheochromocytomas. Analysis identified a heterozygous pathogenic variant in the VHL gene, confirming VHL disease. A comprehensive screening for associated tumors (retinal, cerebellar, renal, neuroendocrine) was negative. The left adrenal tumor remained stable in size, and the patient continued to do well without further treatment after 14 months of follow-up. A conservative monitoring strategy was chosen for the left adrenal mass to preserve adrenal function and avoid long-term steroid dependence, considering its stable radiological appearance and the absence of clinical or biochemical recurrence.

## Discussion

Pheochromocytomas are rare catecholamine-secreting tumors that originate in the adrenal medulla. Paragangliomas and other extra-adrenal tumors of the sympathetic nervous system are classified under paragangliomas and pheochromocytomas [1]. The incidence of these tumors is estimated at 0.46 to 0.8 cases per 100,000 person-years [2]. While most pheochromocytomas are benign, approximately 10% are malignant, 10% are bilateral, and 10% are associated with hereditary syndromes. The average age of diagnosis is around 9.5 years, although cases have been reported in newborns [3].

Pheochromocytomas exhibit unique characteristics in pediatric patients. Approximately half of these tumors are unilateral, while a quarter are bilateral, and another quarter are extra-adrenal (paragangliomas). Extra-adrenal tumors are predominantly located below the diaphragm (85%), with the remaining 15% found in supradiaphragmatic regions, such as the posterior mediastinum [4].

The clinical presentation of pheochromocytomas is highly variable, driven by the effects of catecholamines on various target organs. While adults typically present with the classic triad of headaches, palpitations, and sweating, children often exhibit additional symptoms, including growth arrest, weight loss, polyuria-polydipsia syndrome, and neurological manifestations such as seizures or hemiplegia [4,5]. In our patient, the initial presentation included severe headaches, vomiting, and generalized seizures, complicated by status epilepticus. These symptoms underscore the importance of considering pheochromocytoma in cases of refractory hypertension, especially in pediatric patients.

Diagnosis relies on biochemical evidence of catecholamine overproduction, typically assessed through the measurement of plasma or urinary metanephrines. Plasma-free metanephrines offer the highest sensitivity (97-99%) and are the preferred screening method for patients with a strong clinical suspicion of pheochromocytoma [6]. Localization of the tumor is achieved through imaging techniques, with CT scans being the first-line modality due to their high sensitivity and cost-effectiveness. MRI is particularly useful for evaluating soft tissue contrast and ruling out vascular invasion. Functional imaging, such as meta-iodo-benzyl-guanidine (MIBG scintigraphy) or PET/CT with radiotracers like 18F-FDG, complements anatomical imaging by confirming tumor activity and detecting metastases [1].

Preoperative management is critical to minimize the risk of hypertensive crises during surgery. Alpha-blockers, such as phenoxybenzamine or doxazosin, are commonly used to stabilize blood pressure, with beta-blockers added if tachycardia persists [7]. In our case, the patient was successfully prepared with doxazosin before undergoing a unilateral right adrenalectomy. The decision to perform a partial versus total adrenalectomy in bilateral cases remains a topic of debate. While partial adrenalectomy preserves adrenal function and avoids lifelong steroid dependence, it carries a higher risk of tumor recurrence [8]. In our patient, the unilateral approach was chosen to balance these considerations, and the postoperative course was favorable, with normalization of blood pressure and catecholamine levels.

Approximately 30-50% of pheochromocytomas are associated with hereditary syndromes, such as multiple endocrine neoplasia types 2A and 2B, neurofibromatosis type 1, and VHL disease. Genetic testing is recommended for young patients, those with bilateral or multifocal tumors, and those with a family history of syndromic features [9,10]. In our case, genetic analysis identified a pathogenic variant in the VHL gene, confirming a diagnosis of VHL disease. This autosomal dominant disorder predisposes patients to a variety of tumors, including retinal and central nervous system hemangioblastomas, renal cell carcinoma, and pancreatic neuroendocrine tumors. Early identification of VHL mutations enables proactive surveillance, which is essential for the timely detection and management of associated malignancies [11].

Annual screening is recommended for children with VHL mutations, including clinical evaluation, blood pressure monitoring, and biochemical testing for metanephrines. Abdominal imaging should begin at age 5 to monitor for adrenal and renal tumors. These measures aim to detect pheochromocytomas early and prevent complications such as hypertensive crises or cardiovascular damage [12]. The risk of recurrence in VHL-associated pheochromocytoma remains substantial, especially after partial adrenalectomy. This underscores the importance of maintaining strict, long-term surveillance throughout life, even in asymptomatic patients. Beyond tumor detection, long-term follow-up allows early identification of recurrences and improves patient outcomes. These principles are emphasized in the Endocrine Society guidelines, which recommend lifelong monitoring for all patients with hereditary pheochromocytoma [1].

## Conclusions

Pheochromocytomas, though rare, are a critical cause of secondary hypertension in children. Early diagnosis guided by biochemical testing and imaging is essential to prevent severe cardiovascular complications. This case underscores the importance of considering pheochromocytoma in pediatric patients with refractory hypertension, particularly when presenting with atypical symptoms such as seizures.

Genetic testing is crucial, as up to 50% of pediatric cases are linked to hereditary syndromes such as VHL disease. Identifying these mutations enables proactive surveillance for recurrent tumors and associated malignancies, improving long-term outcomes. In this case, conservative monitoring of the unresected adrenal tumor was chosen to preserve adrenal function, given its stable radiological and hormonal profile. Optimal management of such complex cases requires a multidisciplinary approach involving pediatric endocrinologists, geneticists, surgeons, cardiologists, and radiologists to ensure comprehensive care and tailored surveillance.
